# The story of elimination of visceral leishmaniasis (kala-azar) in India—Challenges towards sustainment

**DOI:** 10.1371/journal.pntd.0013321

**Published:** 2025-08-19

**Authors:** Shyam Sundar

**Affiliations:** Department of General Medicine, Institute of Medical Sciences, Banaras Hindu University, Varanasi, India; Institute of Postgraduate Medical Education and Research, INDIA

## Abstract

The earliest record of visceral leishmaniasis (kala-azar, KA, VL) dates back two centuries from Jessore (now in Bangladesh), with 0.75 million deaths in 3 years. In the 1950s, there was extensive insecticide dichlorodiphenyltrichloroethane (DDT) spray under the aegis of the National Malaria Eradication Program. As a corollary benefit, there was a sharp decline in the incidence of VL due to the reduced prevalence of the vector to extremely low levels, resulting in substantial decreases in the number of KA cases. In the early 1970s, a surge in the number of cases was noted, and since then, it has taken the shape of the current epidemic. In 1990–91, the National Kala-azar Control Program was launched in India, though without much impact due to the diminishing efficacy of treatment with pentavalent antimonial. This was followed by the introduction of highly effective amphotericin B deoxycholate. In 2005, the Kala-azar Elimination Program (KAEP) was launched jointly by India, Bangladesh, and Nepal, with treatment initially with oral miltefosine, succeeded later by single-dose liposomal amphotericin B (AmBisome), and in 2023, India achieved the goal of kala-azar elimination, defined as an incidence below 1 per 10,000 at sub-district (block) level. Patients with post kala-azar dermal leishmaniasis, HIV-VL coinfection, and undiagnosed/untreated VL patients are the human sources for the vector. They may herald an outbreak resulting in the commencement of a new epidemic. Active case detection, early diagnosis, and prompt, complete treatment are required to prevent fresh transmission. Periodic updates of health personnel, community awareness, and continued availability of the theragnostics are important steps for early detection and containment of an outbreak.

## Author summary

After extensive DDT spray in the 1950s for the National Malaria Eradication Programme, the incidence of kala-azar (visceral leishmaniasis, VL, KA) declined remarkably in India. However, in the early 1970s, a resurgence of VL was noticed, which took enormous proportions, and an estimated several hundred thousand cases of VL occurred between the 1970s and 2000s. Unfortunately, the efficacy of the only available anti-kala-azar drug, sodium stibogluconate, declined sharply, and the cure rate plummeted to 35%. Thus, a huge proportion of kala-azar patients were left without any effective treatment. It was a desperate state for a physician like me; amphotericin B deoxycholate came to the rescue, but only for a small proportion of patients because of the logistics involved (unaffordable cost, ~ 5 weeks of hospitalization, high incidence of adverse reactions, etc.). We came across miltefosine, the first oral anti-VL drug, and with a 10-minute VL rapid diagnostic test, the Kala-azar Elimination Programme came to the rescue of the sufferers of this dreaded disease. After a single-dose treatment with liposomal amphotericin B (AmBisome, Gilead Sciences, Foster City, USA) was successfully implemented in this programme, a steep decline in the prevalence of VL occurred. In the year 2023, the goal of the Elimination Programme was achieved in all 633 VL endemic blocks of India. However, being a source of parasites, patients with post kala-azar dermal leishmaniasis, HIV-VL coinfection, and undiagnosed VL, pose a threat for new outbreaks, and a constant vigil is needed for early detection and containment of these.

## Background

Visceral leishmaniasis (VL) is a life-threatening, vector-borne disease that is invariably fatal if untreated. Globally, an estimated incidence of 30000 [1] new cases are reported annually across 99 countries. In 2023, approximately 83% of global VL cases were reported from seven countries: Brazil, Ethiopia, India, Kenya, Somalia, South Sudan, and Sudan [2]. In the Indian subcontinent (ISC), India accounts for the majority of VL cases, and most of the cases are from the state of Bihar. In ISC, VL is caused by *Leishmania donovani* transmitted anthroponotic (human to human) by the sandfly vector *Phlebotomus argentipes.* Although there are reports of dogs transmitting *L. donovani* to sandflies in India, its role in human transmission is yet to be established [3].

**Fig 1:**
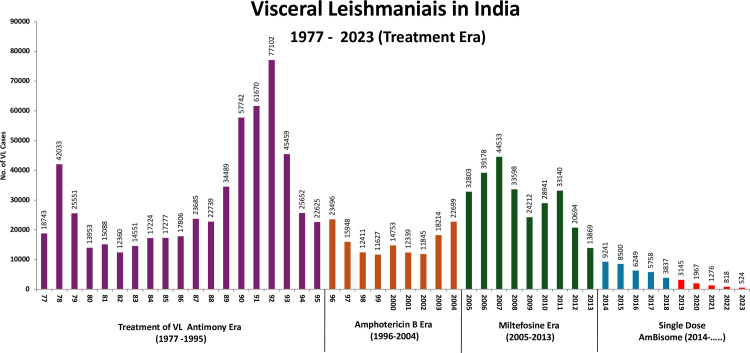
Annual incidence of visceral leishmaniasis in India from 1977 to 2023 with the treatments used (Source of data: National Center of Vector Borne Diseases Control, Government of India).

VL has been present in India for centuries. The earliest documentation of VL in India can be traced back to 1824–1825, when an outbreak of fever in Jessore (now in Bangladesh), known as jwarvikar (“fever disease”), caused approximately 750,000 deaths over 3 years [4]. VL was almost eliminated in India in the 1950s following extensive insecticide spraying under the National Malaria Eradication Program. However, in the year 1974, a resurgence was reported, initially in a small region of North Bihar affecting four districts (Vaishali, Muzaffarpur, Samastipur, and Sitamarhi). After that, VL became endemic across the entire state of Bihar, some parts of the state of Jharkhand, several districts in the state of West Bengal, and the eastern districts of Uttar Pradesh [5,6]. The VL incidence increased, and in the year 1977, the recorded incidence was 18,743, and two peaks in the annual incidences were noted with 42,033, and 77,102 in the years 1978 and 1992, respectively (Fig 1). These were not the real numbers and could be 4.2–8 times more as there was significant underreporting [7,8]. Thus, the actual incidence of VL might have been in several hundreds of thousands during these years.

There were several issues in managing VL in this era; the diagnosis was dependent on the demonstration of parasites in tissue smears from the bone marrow or spleen. However, it was expensive with limited availability, often delaying the diagnosis and subsequently the treatment. Treatment was solely dependent on 30-day parenteral treatment with pentavalent antimony, sodium stibogluconate (Sb^V^). There was widespread resistance to Sb^V^, and hence, a vast majority of VL patients with active disease remained roaming in the community with a rich source of parasites, continuously propagating the transmission of the disease.

The Government of India launched a centrally sponsored Kala-azar Control Programme in 1990–91. The programme was intensified during the year 1991, resulting in a reduction of morbidity and mortality to 22,625 cases and 277 deaths in 1995 in comparison to a peak incidence of 77,102 cases and 1,419 deaths reported during 1992 [9]. However, unfortunately, during these years (the 1990s to early 2000s), the efficacy of the pentavalent antimonial (sodium stibogluconate; Sb^V^) plummeted to 35–36% [10,11].

The only alternative during these years, was amphotericin B deoxycholate (AmB), though highly effective (cure rate >98%), the safety profile of AmB is not good, and 5–6 weeks of hospitalization is necessary for 15–20 intravenous infusions (daily or alternate days) with continuous laboratory monitoring [12,13]. With these handicaps, there was a massive shortage of hospital beds for such a large number of patients. Initially, AmB was primarily reserved for patients with Sb^V^ treatment failure. In August 2000, the National Expert Committee on Kala-azar recommended the use of AmB as the primary treatment for VL endemic areas where the rate of Sb^V^ treatment failure was greater than 10%. However, because of the reasons stated above, the use of AmB as a first-line treatment did not succeed. Dependence on ineffective Sb^V^ continued.

Meanwhile, oral miltefosine (MF) became available as an effective antileishmanial drug and was approved for the treatment of VL in India in 2002 [12,14]. ‘At that time the rK39 rapid immunochromatographic test became available, allowing a presumptive diagnosis based on the combination of specific symptoms and presence of antibodies in a capillary blood sample’. [15], complemented by the ease of administration of oral MF treatment, both of these new diagnostic and treatment tools were easily deployable in the most remote health facilities in India as well as in Nepal and Bangladesh. Thus, the stage was set for an aggressive KA control program, in 2005, a joint Kala-azar Elimination Programme (KAEP) was launched by the Governments of India, Nepal, and Bangladesh to reduce the annual incidence of VL to less than 1 case per 10,000 population at the sub-district level by 2015 [16].

The key strategies adopted for the National KAEP were as follows [17]:

1. Early diagnosis and complete case management
2. Integrated vector management (IVM) and vector surveillance
3. Supervision, monitoring, surveillance, and evaluation
4. Strengthening capacity of human resource in health
5. Advocacy, communication, and social mobilization for behavioral impact and inter-sectoral convergence
6. Programme management

### Progress made in VL elimination initiatives

**Fig 2:**
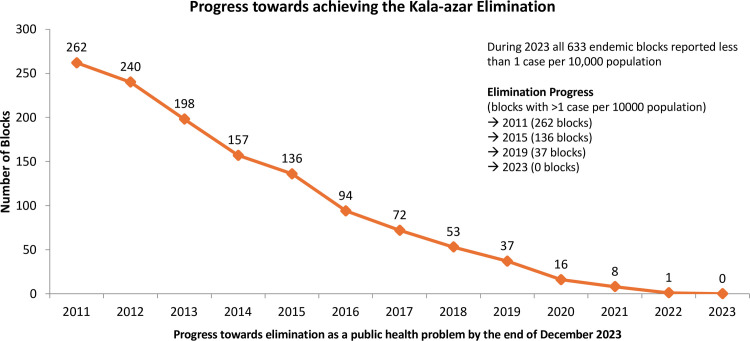
Progress towards achievement of the kala-azar elimination from 2011 to 2023 (Source of data: National Center of Vector Borne Diseases Control, Government of India).

In 2005, when the VL elimination initiatives started, an estimated 165.4 million people in India were at risk of leishmania infection, and 34,803 active VL cases were reported from 633 public health center (PHC) blocks across four states that were endemic (Figs 1 and 2). This included 33 districts and 458 blocks of Bihar; 4 districts and 33 blocks of Jharkhand; 11 districts and 120 blocks of West Bengal; and 6 districts and 22 blocks of Uttar Pradesh. The reported VL case load in the region decreased significantly, from over 34,803 cases in 2005 to fewer than 8,243 cases in 2015, and further declined to 524 cases in 2023 (Fig 1). Similarly, the number of endemic blocks attaining the elimination target of less than 1 in 10,000 decreased from 633 in 2005 to 136 in 2015, 8 in 2021, and ultimately reached 0 by 2023 (Fig 2). The last block that achieved the elimination target was in Pakur district, Jharkhand. Through the VL elimination initiatives, the number of VL cases in India significantly declined, and by 2023, the South East Asia Region of WHO (India, Bangladesh, and Nepal) reported only 6% of cases reported from the world [18,19].

In 2023, the WHO certified Bangladesh as the first country to be validated for VL elimination as a public health problem. In December 2023, India also achieved the elimination threshold in all implementation units (Fig 2) and transitioned into the consolidation phase of sustaining elimination for a minimum of three consecutive years before submitting a dossier to the WHO for validation. In 2023, the rates in two implementation units in Nepal were above the elimination threshold. Due to the success of the elimination initiative in the WHO-SEAR, which now has 6% of the worldwide burden and the fewest cases since the launch of the elimination initiative in 2005 [20].

### Challenges and threats to sustainment of VL elimination

As India has achieved the goal of elimination, there are challenges to prevent new outbreaks, as there are lurking dangers of the restart of the epidemic due to persistent parasites, in the form of human reservoirs, circulating in the community. Primarily A—post kala-azar dermal leishmaniasis and B—HIV-VL coinfection.

The asymptomatic infected individuals, though speculated to play a part in the transmission, however, in a xenodiagnosis study involving 184 subjects with high antileishmanial antibody titers, none of the sandflies could be infected by any individual from this cohort [21]. Thus, it was concluded that these individuals may play only an insignificant role in the transmission of VL. Active case detection with early initiation of treatment could minimize the contribution of patients with unrecognized illness in the disease transmission.

There have been reports of VL from hilly states of India like Himachal Pradesh and Uttarakhand. From Nepal also, outbreaks of VL have been reported from highlands, though earlier, only a few districts from the Southeast region (low land area) were endemic for VL. These changes in endemicity do hint at the effects of climate change, with vectors being able to migrate to higher altitudes.

### Early diagnosis and treatment

There is clear evidence that VL tends to cluster within household members, close contacts, and neighborhood contacts. This phenomenon suggests that VL patients may play a significant role in transmission. It has been proven through xenodiagnosis studies that, indeed, these patients do transmit the infection to the vectors quite proficiently. In a study from India, 60 (78%) of 77 patients with VL transmitted parasite infection to the sandflies [21]. In another study from Bangladesh, 66.7% of VL patients infected the sandflies. [22]. These findings underscore the importance of early diagnosis and effective treatment of VL. With the introduction of rapid diagnosis of VL by rK39 immunochromatographic strip test, early detection of VL has become possible, though the rK39 rapid diagnostic test is highly specific for the anti-K39 antibody detection [15], with significantly decreased incidence of VL, a seropositive subject should be looked for clinical/laboratory features of VLIf needed, parasitological or PCR confirmation of presumptive diagnosis by rapid test can also play a role.

This could be an important step in restricting the transmission. Similarly, and more essentially, to break the transmission chain effectively and early, another important step will be to start effective treatment immediately, with an appropriate follow-up, till a definitive cure is achieved. Single-dose liposomal amphotericin B (LAmB) treatment has been a great success in the treatment of VL [23], and its deployment in the KAEP has been the most important factor in the achievement of the elimination goal in India and Bangladesh. Being with an impeccable safety profile, its use in the treatment of VL is possible even at the most peripheral health set-up in the endemic areas of ISC. Its prompt use in the treatment of VL will have a pivotal role in successfully restricting the transmission of this disease.

Although LAmB, used in the KAEP, is available as a free donation from the manufacturer Gilead Sciences, Foster City, US, it is unaffordable and expensive in the open market (60 US$ for a 50 mg vial) in India. The good news is that now indigenous liposomal Amphotericin B formulations are available at much lower price (12–14 US$/50 mg), though equivalence studies need to be done to validate the safety and efficacy profile of these new formulations. Unfortunately, with a steep decline in the number of VL patients, it will be impossible to conduct such studies.

### Post kala-azar dermal leishmaniasis (PKDL)

**Fig 3:**
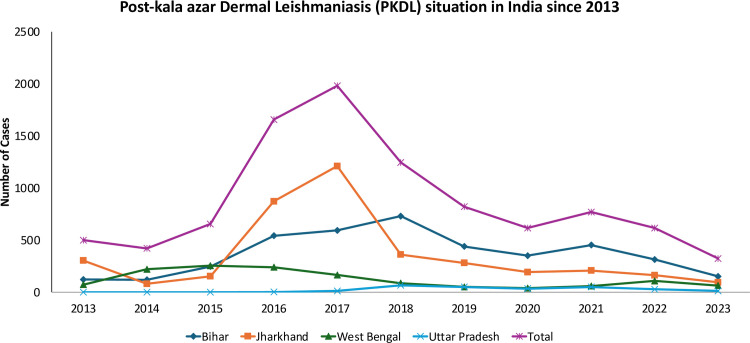
Post kala-azar dermal leishmaniasis (PKDL) situation in India during 2013–2023 (Source of data: National Center of Vector Borne Diseases Control, Government of India).

In India, within 1–3 years or longer post-treatment for VL, a small percentage (5–15%) of individuals develop skin eruptions such as nodules or papules, a condition termed PKDL [24]. The primary importance of PKDL derives from its role as an infection reservoir [21]. PKDL patients are not systemically ill and may remain untreated for years. Treatment requires inordinately long courses of sodium stibogluconate (SSG) [25] or AmB [26] and, more recently, of MF [27]. These patients are human reservoirs for the parasites and may initiate fresh outbreaks. There are reports of VL outbreaks attributed to patients of PKDL. Cases of persistent PKDL in West Bengal have been implicated as the origin of the resurgence of VL in that region [28,29]. A large number of patients with PKDL (Fig 3) can have the lesion for years without seeking treatment and, thus, could be infectious for a longer time than patients with active VL. If untreated, the disease persists as there is no self-cure, unlike Sudanese PKDL. Patients with nodular PKDL were more infectious to sandflies than patients with macular PKDL, both in the proportion of flies infected and in the number of parasites transmitted. Interestingly, during a xenodiagnosis study, sandflies fed on the normal skin of patients, with only macular PKDL, were able to pick up leishmania infection [21]. These findings underscore the important role that patients with PKDL play in the transmission of leishmanial infection, irrespective of the morphology of the lesions.

Except for the aesthetic concerns, there are no physical disabilities beyond cosmetic disfigurement. In a significant proportion of patients, these lesions are not of major concern. Thus, most patients are reluctant to undergo treatment. Sometimes, severe cosmetic disfigurement, with associated stigma, drives patients to seek treatment. ‘Unfortunately, treatment regimens for PKDL are far from satisfactory. Previously, inordinately prolonged and toxic treatments were recommended for its management. The standard recommendation with Sb^V^ treatment of PKDL was intramuscular injections in doses of 20mg/kg daily for 120 days, and in case of inadequate response, the treatment could be extended up to a maximum of 200 days [25,30]. In case of Sb^V^ failure, three to four 20-day courses of intravenous infusion of AmB (1mg/kg) is recommended with a treatment-free interval of 20 days [26]. In the WHO recommendations of the year 2010, a 12-week treatment of oral MF is recommended [31]. Each one of these PKDL treatment regimens, in addition to being protracted, is associated with unacceptable toxicities. Sb^V^ can cause serious and occasionally fatal cardiotoxicities [11]. With AmB, high incidence of infusion reactions, frequent nephrotoxicity, hypokalemia, and cardiotoxicity are limiting factors. With prolonged MF treatment of PKDL patients, ocular toxicity (partially reversible) [32] is a serious concern; potential teratogenicity of MF rules out its use in pregnancy, and further, it necessitates contraceptive practices for the duration of the treatment and an additional 5 months. In a nutshell, currently, available PKDL treatment regimens are far too prolonged, toxic, expensive, and unacceptable; incomplete treatment and frequent dropouts limit successful therapy. However, a recent report of treatment of PKDL with liposomal amphotericin B (LAmB), at a total dose of 20 mg/kg in five divided doses administered over 2 weeks, led to a final cure of 83% of patients at a 24-month follow-up. This short-duration 2-week therapy is patient-friendly, which will be more acceptable to PKDL patients [33].

The unfortunate part about PKDL is that interest in this important entity has been much lower from academia, industry, and national control programmes of the ISC as well as the World Health Organization (WHO). However, since 2011, WHO has been strengthening the countries’ surveillance of PKDL, In the last decade (years 2014–2023), a total of 9,108 cases have been reported from India [19]. There is an urgent need to implement the recently described 2-week treatment [33] and further research to find an even shorter, safe, and affordable (preferably oral) regimen for the treatment of PKDL.

### HIV-VL co-infection

In India, the prevalence of HIV among VL patients has increased from 0.88% in 2000 to 2.18% in 2006 [34–36]. In 2022, WHO reported that up to 5–7% of patients with VL in India were identified with HIV co-infection, marking the highest rate in South Asia [37]. It has been shown that HIV-VL co-infected patients transmit the *L. donovani* infection very efficiently. These patients remain infectious for long periods, as they are unable to clear the infection completely and keep relapsing with signs and symptoms of VL. Due to their potentially high parasitic load and increased tendency to relapse, HIV-VL co-infected patients are capable of transmitting the disease very efficiently. HIV-VL coinfected patients are highly infectious to sandflies in the endemic areas in India [38] for extended periods. In a xenodiagnosis study, 93% (13/14) of the VL-HIV group successfully transmitted the leishmania infection to the sandflies and were three times more infectious than those who had VL without HIV. Thus, HIV-VL coinfected patients are a big threat to maintaining the status of elimination. It is important that these patients are searched actively and treated strenuously for both diseases. Fortunately, for both of these diseases, free treatment is available in India. Prevention of VL relapses is vital, and this can only be done by effective antiretroviral treatment with successful antileishmanial therapy. It is also recommended that prophylactic monthly AmB or liposomal amphotericin B therapy be administered till a CD4 + count of ≥350 is achieved [39].

### Vector control and environmental measures

The indoor resting behavior of sandfly (*P. argentipes*) makes them a suitable target for control by indoor residual spraying (IRS) with insecticides. As a result, control of malaria with DDT starting in 1950s immensely benefitted VL control in the ISC from 1953 to 1962, IRS undertaken by the Indian National Malaria Program using DDT for malaria control had immense beneficial side effect of controlling sandflies and significantly reducing the number of VL cases in the ISC [40,41]. This collateral benefit led to the adoption of IRS with DDT by the Indian VL elimination program as the main focus for *P. argentipes* control. After the resurgence of VL in the ISC and the launch of KAEP, DDT was used to eliminate the VL vector from the country [42]. Sandflies were known to be susceptible to DDT until the first report of resistance in *P. argentipes* in 1993 [43]. Given the paramount importance of IRS to the VL control effort, the National Vector Borne Disease Control Programme changed to an effective insecticide, the synthetic pyrethroid alpha-cypermethrin (5%) as an alternative in the second phase of IRS and altered the mode of application to hand compression pumps to improve the quality of IRS delivery. Continuous monitoring of the insecticide resistance should be in place for timely intervention and course correction in case the emergence of insecticide resistance is detected.

Sustained vector control is an important component to bring down/stop the transmission of VL. IRS should be continued with the same vigour and zeal as in the pre-elimination era. Insecticide selection should be judicious, with constant monitoring of their sensitivity. Implementing an IVM approach, which combines biological, chemical, and environmental control methods, can enhance the effectiveness of vector management efforts.

IRS activities are resource incentive; other methods of vector control are also vital. One of the most important steps is modifying the environment and abrogating the breeding sites. Financial assistance for the conversion of straw or mud dwellings into plastered brick houses for removing the cracks/crevices, the sites for sandfly breeding. Animal shelters should be modified to prevent the sites for vector breeding, etc. Ultimately, instead of blanket spray, focal spray around the areas of an outbreak could be a viable alternative.

As the elimination goal is achieved, the political and donor interests are bound to decline; however, strengthening the Primary Health Centers by integrating the resources set aside for management of various vector-borne diseases like VL, malaria, and filaria is likely to be the way forward. The close collaboration between India, Nepal, and Bangladesh, as existed in the pre-elimination era, should continue. WHO–South East Asia Regional Office (SEARO) must continue its important role through the Regional Technical Advisory Group (RTAG) in coordinating the efforts for elimination, which is very important.

### Surveillance

Active case detection for early recognition of VL and PKDL; and early diagnosis and treatment are of paramount importance in the recognition and containment of new outbreaks. Community education for the two leishmanial diseases should be continued for continued awareness. Periodic refresher workshops for healthcare providers are extremely important for continued updating of knowledge. Continued availability of appropriate diagnostic and treatment facilities for both VL and PKDL must be ensured during the post-elimination phase.

### Role of various stakeholders in KAEP

Although the KAEP was largely funded by the Government of India, partnerships with various global agencies played an important role in the success of the program. WHO headquarters and its SEARO formed an RTAG, which helped in policy making, and if needed, course corrections were advised periodically. Bill and Melinda Gates Foundation’s financial, technical, and research support has been important in the success of the KAEP in the ISC. Other agencies like the Foreign, Commonwealth and Development Office (FCDO), UK, the National Institute of Allergy and Infectious Diseases, and National Institute of Health, US, also supported the program, funding important research and program implementation.

Academia played a very important role in developing tools for elimination, early diagnosis with rK39 RDTs played a crucial part resulting in the quick identification and elimination of the VL, similarly clinical development of oral MF provided an important alternative to Ampho B/ Sb^V^ treatment, these two innovations were vital which made the possibility of the very concept to KAEP in the ISC. Later in the program, single-dose LAmB (AmBisome) treatment, which was introduced in the program in the year 2014, played the most important role in achieving the goal of KAEP.

The National Center for Vector-Borne Disease Control (NCVBDC) played an important role in the implementation of national health strategies and allocated domestic funding. Strengthen disease surveillance, ensure early diagnosis, and provide free treatment. The state health machinery executed these tasks. Similarly, vector control implementation with the IRS, surveillance, and case management, with training of healthcare workers at various levels for early case detection and treatment, was guided by the central agency and executed by the state governments. NCVBDC has also been maintaining the national register of kala-azar, PKDL, and HIV-VL patients and has done an impeccable job since the onset of the VL epidemic in the early 1970s till date.

Non-governmental organizations like PATH (Program for Appropriate Technology in Health), DND*i* (Drugs for Neglected Diseases initiative), and CARE India (Cooperative for Assistance and Relief Everywhere) collaborated with the governmental machinery for the success of KAEP. PATH assisted in streamlining logistics and distribution of diagnostics and treatment drugs and training of health care personnel. CARE did community mobilization and developed the Kala-azar Management Information System (KAMIS) database, which was extremely helpful in reporting and monitoring the progress of KAEP in real time. NCVBDC, with the help of state machinery, is maintaining the KAMIS database now. DND*i* supported several clinical trials in kala-azar and PKDL.

### Limitations

Diagnosis has been based on antibody detection, which has several pitfalls. Antibodies, being persistent for several years after cure, do not offer a test of cure and are of no use in the diagnosis of relapses. There is a need for rapid antigen detection tests which will be devoid of the demerits enumerated above.

Single-dose LAmB treatment was key to the success of KAEP, but this is dependent on the donation program, which was the result of WHO negotiations with Gilead Sciences, and it continues till the end of 2025. It is important to assess the generic LAmB’s safety and efficacy in the treatment of VL, to ensure the ready availability of the drug for sporadic cases. Low numbers of patients is a severe handicap in the successful development of new drugs for clinical use. It remains crucial to develop new safe and effective oral antileishmanial drugs, obviating the need of infusions. A cold chain is needed for the transport and storage of LAmB, and this is a great challenge in the endemic areas of ISC, where room temperatures are high during most of the year. The drugs that can be stored at ambient temperatures are sorely needed.

Another important limitation is the unsatisfactory treatment of PKDL. The current recommendation is treatment with oral MF for 84 days. In addition to being of an unduly prolonged duration, its potential teratogenicity, non-compliance, and significant ocular toxicity make this regimen unsuitable for widespread deployment in the community. There is an urgent need to find a suitable, short, and safe treatment for PKDL in the ISC.

As the number of VL and PKDL patients has gone down significantly, it will be a challenge to cater to these patients, who are sporadically located in various endemic districts. Regional depots should be created at strategic locations easily accessible to the entire region. Availability of rK39 rapid diagnostic test should be ensured in the health facilities in all endemic regions/districts even after reaching the elimination target, for early detection and treatment.

## Conclusions

KAEP has been a successful endeavor with the achievement of the goal of the elimination of VL in India. Early diagnosis by rK39 rapid test and the availability of an oral drug, MF, were tailor-made for this endeavor, compared to the dependence on invasive and risky parasitological diagnosis, and toxic and ineffective therapy. Later, the introduction of single-dose L-AmB complete treatment further accelerated the pace of success. These tools were the major difference that separated this effort from earlier unsuccessful attempts to control and eliminate this dreaded disease. The new tools also limited the transmission chain to a low level, and the number of patients decreased at a greater pace. The strong political support was of great value, and the Central and State governments worked in tandem. WHO (Geneva and SEARO) also played a stellar role; several external not-for-profit organizations joined hands. Post-elimination, it is a challenge to keep the incidence of VL at or below the elimination goal. Two major threats to sustain the achievement of KAEP are PKDL and HIV-VL co-infection. Both conditions are human reservoirs of the parasite. In these ailments, patients either do not want the treatment, as in PKDL, and in HIV-VL coinfection, patients keep relapsing till the immune status improves with successful antiretroviral treatment. On the other hand, the treatment regimens of PKDL remain unsatisfactory. These two remain a threat for new outbreaks as low levels of community transmission increase the susceptible population with low immunity and thereby increase susceptibility. Active case detection for early recognition of VL and PKDL, and early diagnosis and treatment is of paramount importance. Sustained vector control is another important component. IRS should be continued with the same vigor and zeal as in the pre-elimination era. Strong political commitment with continued availability of the resources, continued vector control, active case detection not only of VL but also PKDL and prompt and complete treatment remain important to sustain the achievements of KAEP.
